# Bathing

**Published:** 1869-08

**Authors:** 


### BATHING.

We perfectly detest sea bathing; we do not believe that it ever did anybody any good beyond what could have been attained at half the cost and trouble at home. Besides, men went in bathing at Long Branch, Cape May and Absecom last year, one at each place, and they hav'n't yet returned. It is a great deal easier to avoid drowning than to be restored after one is "dead as a door-nail," if any one knows how dead that is. The indelicacies, and positive indecencies connected with sea bathing at our fashionable resorts are disgusting to all persons of true culture. But as some differ from us, we will make some suggestions for their benefit.

1. Bathe where nobody else can see you.

2. Don't, for goodness sake, bathe in borrowed garments.

3. Don't bathe in any garments at all, if your object is cleanliness.

4. Sick or well, don't remain in the water longer than ten minutes, and be in active motion all the time.

5. On leaving the water wipe dry, dress as soon as possible, and on reaching your apartment lie down until thoroughly rested.

6. Never bathe but once a day, even if in perfect health; then you don't need it except as an amusement.

7. Never bathe on a full stomach; before breakfast is the safest, most agreeable and most healthful hour for taking a bath.

8. Walk from the bath to the house; riding in a wind has often caused inflammation of the lungs or troublesome colds of long duration.

